# Optimal Upfront Treatment in Surgically Resectable Pancreatic Cancer Candidates: A High-Volume Center Retrospective Analysis

**DOI:** 10.3390/jcm10122700

**Published:** 2021-06-18

**Authors:** Sarah Maloney, Malinda Itchins, Jennifer Arena, Sumit Sahni, Viive M. Howell, Sarah A. Hayes, Anthony J. Gill, Stephen J. Clarke, Jaswinder Samra, Anubhav Mittal, Nick Pavlakis

**Affiliations:** 1 Faculty of Medicine and Health Sciences, Northern Clinical School, The University of Sydney, Sydney, NSW 2065, Australia; mitchins@gmail.com (M.I.); Jennifer.Arena@health.nsw.gov.au (J.A.); sumit.sahni@sydney.edu.au (S.S.); viive.howell@sydney.edu.au (V.M.H.); sarah.hayes@sydney.edu.au (S.A.H.); Anthony.Gill@health.nsw.gov.au (A.J.G.); stephen.clarke@sydney.edu.au (S.J.C.); jas.samra@bigpond.com (J.S.); anubhav@mittal.com.au (A.M.); nick.pavlakis@sydney.edu.au (N.P.); 2 Bill Walsh Translational Cancer Research Laboratory, Kolling Institute, The University of Sydney, Sydney, NSW 2065, Australia; 3 Department of Medical Oncology, Royal North Shore Hospital, St. Leonards, Sydney, NSW 2065, Australia; 4 Upper Gastrointestinal Surgical Unit, Royal North Shore Hospital, St. Leonards, Sydney, NSW 2065, Australia; 5 Cancer Diagnosis and Pathology Group, Kolling Institute, The University of Sydney, Sydney, NSW 2065, Australia; 6 NSW Health Pathology, Department of Anatomical Pathology, Royal North Shore Hospital, St. Leonards, Sydney, NSW 2065, Australia

**Keywords:** resectable, pancreatic, cancer, neoadjuvant, surgery, biomarkers

## Abstract

Pancreatic adenocarcinoma is a devastating disease with only 15–20% of patients resectable at diagnosis. Neoadjuvant chemotherapy for this cohort is becoming increasingly popular; however, there are no published randomized trials that support the use of neoadjuvant chemotherapy over upfront surgery in resectable disease. This retrospective cohort analysis was conducted to compare both treatment pathways and to identify any potential prognostic markers. Medical records from one large volume pancreatic cancer center from 2013–2019 were reviewed and 126 patients with upfront resectable disease were analyzed. Due to a change in practice in our center patients treated prior to December 2016 received upfront surgery and those treated after this date received neoadjuvant chemotherapy. Of these, 86 (68%) patients were treated with upfront surgery and 40 (32%) of patients were treated with neoadjuvant chemotherapy. Our results demonstrated that patients treated with upfront surgery with early-stage (1a) disease had a longer median OS compared to those treated with neoadjuvant chemotherapy (24 vs. 21 months, *p* = 0.028). This survival difference was not evident for all patients (regardless of stage). R0 resections were similar between groups (*p* = 0.605). We identified that both tumor viability (in neoadjuvant chemotherapy-treated patients) and tumor grade were useful prognostic markers. Upfront surgery for certain patients with low volume disease may be suitable despite the global trend towards neoadjuvant chemotherapy for all upfront resectable patients. A prospective clinical trial in this cohort incorporating biomarkers is needed to determine optimal therapy pathway.

## 1. Introduction

Pancreatic ductal adenocarcinoma (PDAC) continues to have a poor prognosis with 9% of patients alive at five years [1]. Only 15–20% of patients at diagnosis are considered to have resectable disease and the standard treatment for these patients includes upfront surgery (UFS) followed by chemotherapy in the adjuvant setting [2]. Despite recent advances in systemic therapy, the cure rate in early-stage disease remains low with five-year survival in margin and node-negative disease of only 30% [3]. Increases in survival rates are impeded in part due to deliverability of these chemotherapeutic agents post-surgery with up to 30% of suitable patients not receiving their planned chemotherapy secondary to surgical morbidity [4].

These early-stage resectable patients comprise of two cohorts. Borderline resectable (arterial: contact with common hepatic artery, superior mesenteric artery or celiac axis artery ≤180 degrees; or venous; contact with superior mesenteric vein or portal vein >180° or ≤180° with contour abnormality) and upfront resectable disease (no arterial contact and tumor contact ≤180° with the superior mesenteric or portal vein) [5].

Although initially used for locally advanced pancreatic cancer with the view to downstage disease to make surgical resection possible, there has been an increase in the use of neoadjuvant chemotherapy (NAC) over UFS in borderline resectable candidates and the success of this in the borderline setting has led to national comprehensive cancer network (NCCN) guidelines recommendation that NAC should be first-line therapy in borderline resectable patients [5,6,7]. The evidence for NAC in the resectable cohort (excluding borderline disease), is not as well established. Randomized controlled trials (RCTs) from Casadei et al. and Golcher et al. comparing UFS with NAC were ceased prematurely due to slow accrual [8,9]. At present two RCTs, PREP-02/JSAP-5 and PREOPANC, are underway to help answer this question. Preliminary results from the PREP-02/JSAP-05 trial reveal an improved median overall survival (OS) in patients receiving NAC (gemcitabine and S1) at 36.7 months compared to UFS at 26.6 months) [10]. While these results provide some optimism on the use of NAC in this population, it must be noted that the chemotherapy combination of gemcitabine and S1 has had limited success in a Caucasian population and is infrequently used due to the success of more intensives regimens such as 5-fluorouracil, irinotecan and oxaliplatin (folfirinox) [11]. PREOPANC-1, another RCT comparing NAC (gemcitabine) plus radiotherapy to UFS in patients with both resectable and borderline disease demonstrated no survival benefit in preliminary results in either group [12]. Both trials have not been completed and final results including subgroup analysis are yet to be published.

At our center, NAC became standard of care for both borderline and resectable patients that were considered chemotherapy candidates from 2016 onwards.

The lack of completed randomized controlled trials comparing UFS to NAC in the upfront resectable setting has led to a clinical equipoise. A concern from clinicians in favor of upfront resection is that the delay to surgery from neoadjuvant chemotherapy may result in disease recurrence beyond resectability and hence render a patient “incurable”. Conversely, the appeal of NAC is that it provides exposure of possible micro-metastatic cells to cytotoxins early and increases the chances of a margin negative surgical resection [13].

In recent years, interest in inherent tumor biology or “biomarkers” as an independent determinant of treatment response and survival has led to extensive genomic and proteomic studies [14]. These studies play a crucial role in assisting clinicians to personalize upfront treatment sequencing, a feat yet to be achieved in this disease. There has also been recent interest in the anatomical location of the primary tumor whether it originates in the head of the pancreas versus the body/tail. Pancreatic body/tail lesions make up a small proportion of all new diagnoses, however tend to behave more aggressively than head tumors [15]. In part, this is due to the anatomical location as tail tumors tend to result in fewer symptoms, however, more recently it is understood that they serve as an independent predictor of tumors of the same stage [16].

In light of the current paucity of evidence, we conducted a retrospective cohort analysis to compare outcomes in patients who received NAC with a retrospective patient cohort in the same center who received traditional UFS.

The aims of this study were to (1) determine if our patient cohort demonstrated an improvement in recurrence-free (RFS) or overall survival (OS) for NAC compared to UFS; (2) establish if administration of NAC resulted in improved surgical outcomes; and (3) identify if biological or imaging markers commonly reported in the literature have a role in prognosis or prediction of chemotherapy response.

## 2. Materials and Methods

### 2.1. Data Acquisition and Cohort Details

From December 2013 to January 2019 patients with resectable disease were identified using prospective chemotherapy and surgical databases, imaging reports, multi-disciplinary team (MDT) discussions and surgeon review. In this cohort, only upfront resectable patients were included. Patients with borderline disease were excluded.

The decision as to upfront treatment (either upfront surgery (UFS) or neoadjuvant chemotherapy (NAC)) was based on time of diagnosis. Due to the success of NAC in the borderline and locally advanced setting, from December 2016 onwards NAC replaced upfront surgery (UFS) as standard of care at our center [17]. In our cohort patients treated prior to this date received UFS and patients after this date were treated with NAC. Patients after December 2016 that were not chemotherapy candidates received upfront surgery.

All surgeries were performed at two hospital sites (Royal North Shore Hospital and North Shore Private Hospital) as part of the same pancreatic cancer center by two specialized pancreatic cancer surgeons (JS and AM). As this is a tertiary referral center, patients were referred from a diversity of metropolitan and regional locations; however, the majority of chemotherapy regimens were given within the one local health district. The choice of regimen and duration of chemotherapy was based on MDT consensus recommendation in collaboration with treating clinicians. Each patient was discussed at our MDT meeting at the time of diagnosis and again during their treatment.

All patients underwent baseline investigations which included: baseline carbohydrate antigen 19-9 (CA 19-9), computerized tomography of the chest, abdomen and pelvis (CT-CAP) as well as endoscopic ultrasound (EUS) guided biopsy for histological diagnosis. Resectability and staging were based on radiological imaging. Fluro-deoxyglucose-positron emission tomography (FDG-PET) scans were performed as per MDT recommendation and were subject to patient preference (due to cost). Staging laparoscopy and peritoneal washings were used as an adjunct pre-operatively in UFS and pre-chemotherapy in NAC patients to rule out peritoneal or radiologically undetectable metastatic disease. For patients undergoing NAC, CA 19-9 and CT scans were repeated preoperatively. RECIST criteria was used for sequential imaging [18]. FDG-PETs were repeated preoperatively in NAC patients and a cut-off of SUVmax of 5 was used to delineate higher and lower uptake for both upfront and pre-operative scans.

Surgical complication rates, margins, venous resection rates, vascular invasion and perineural invasion were obtained. Complications were documented for type and grade of severity (1–5) using the Clavien-Dindo classification [19]. Surgical margins (microscopic) were reported for all tumors. As per guidelines, margins were considered negative if the tumor was >1 mm away from the closest surgical margin (R0) or positive if the tumor was <1 mm from the closest surgical margin (R1) [20].

Histological tumour grade and stage were measured by a gastrointestinal specific pathologist using TNM 8th system [21]. Tumor viability was reported for patients who received NAC as the percentage of the original tumor volume present in the surgical specimen and ranged from 0% (no residual tumor) to 100% (no destruction of the original tumor) viable [22]. This was analyzed as continuous data, due to the lack of consensus agreement within the academic community for use of one validated grading tool.

Potential prognostic and predictive markers of interest were identified through literature, prior to analysis [23]. These markers were included and their roles in prognostication and prediction of chemotherapy response were evaluated.

### 2.2. Statistical Analysis

Patients were analyzed according to the initial treatment plan (NAC vs. UFS) regardless of what treatment they received. RFS and OS were analyzed using Cox proportional hazards or Kaplan-Meier (log-rank test) where appropriate. Prognostic factors were assessed using multivariate and univariate analysis by cox-hazards ratio. Univariate analysis using the chi-squared test was performed on categorical data and spearman’s rank analysis was performed on non-parametric variables to assess correlation. One-way ANOVA was performed to assess baseline differences in continuous variables between groups. Statistical analysis was performed using SPSS (IBM Corp, Armonk, NY, USA). *p* values < 0.05 were considered statistically significant.

### 2.3. Institutional Ethics

Institutional ethics approval for this study was obtained (HREC/16/HAWKE/105). Written consent was obtained in select patients where possible, however, was not required as it was using historical databases [24]. This study was conducted according to the national statement on ethical conduct in human research.

## 3. Results

**Figure 1:**
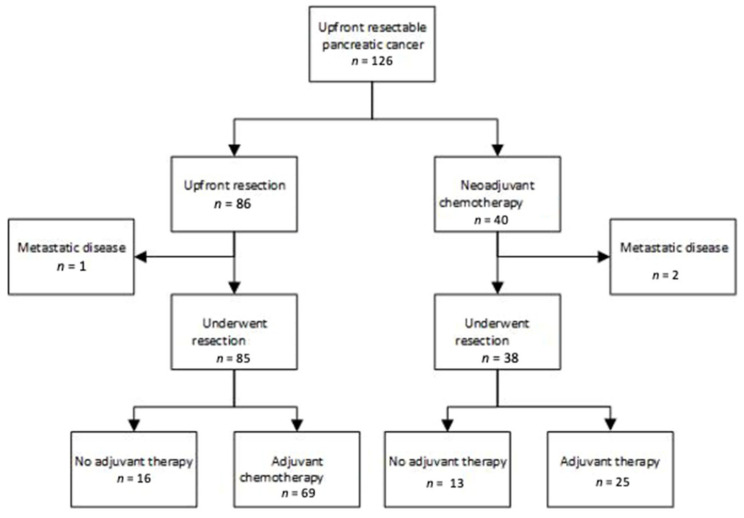
Treatment pathways for patients diagnosed with resectable pancreatic cancer.

Baseline characteristics of the patient cohorts are shown in Table 1. From December 2013 until January 2019, 126 patients from our center were classified as resectable and underwent initial treatment either with neoadjuvant chemotherapy (NAC) (*n* = 40) or upfront surgery (UFS) (*n* = 86) (Figure 1). The median age for all patients was 69 (41–90) years and 58% of patients were male. There were no significant differences in baseline characteristics in the two cohorts including patient age, gender and stage. Staging laparoscopy and peritoneal washings were performed in 45/86 patients (52%) undergoing UFS prior to surgery and in 35/40 (88%) of patients in the NAC cohort prior to chemotherapy commencement. There were significantly more patients in the UFS surgery group with body/tail tumors 23/86 (27%) than in the NAC cohort 2/40 (5%) (chi squared *p* = 0.004).

Neoadjuvant chemotherapy regimens and duration were heterogeneous and are shown in Appendix A Table A1. As all patients were considered resectable (borderline patients were not included), routine chemoradiotherapy was not given. Thirty-eight of the 40 patients proceeded to surgery with two progressing beyond resectability. One patient in the upfront resectable cohort was found to have metastatic disease and surgery did not proceed.

### 3.1. Survival

**Figure 2:**
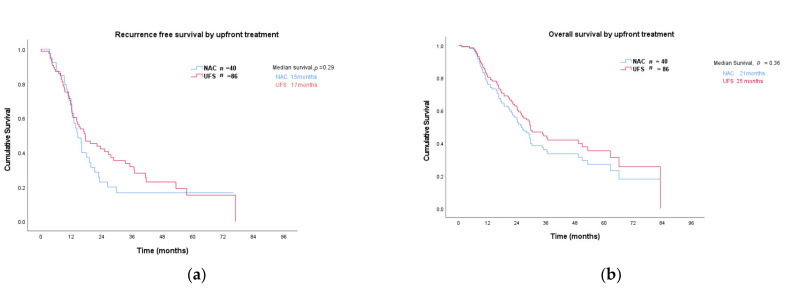
(**a**) Recurrence free; (**b**) overall survival: NAC—neoadjuvant chemotherapy; UFS—upfront surgery.

After a median follow up 52 months, recurrence-free (RFS) and overall survival (OS) of NAC versus UFS patients were evaluated. Survival was calculated using time at diagnosis as this was closer to treatment commencement for both groups. At the time of reporting 31/40 (78%) of NAC and 60/86 (70%) of UFS had recurred and 24/40 (60%) of NAC and 49/86 (57%) of UFS had died. There was no significant difference in median RFS in the UFS compared to NAC group at 17 (0.3–77) months and 15 (3–76) months, respectively, (hazard ratio (HR) 0.79 (0.51–1.2), *p* = 0.29; Figure 2a). Similarly, no difference in median OS was observed between these two groups at 25 (6–83) months in the UFS group compared to 21 (4–76) months in the NAC cohort (hazard ratio (HR) 0.79, *p* = 0.357; Figure 2b).

### 3.2. Upfront Radiological Stage and Location

**Figure 3:**
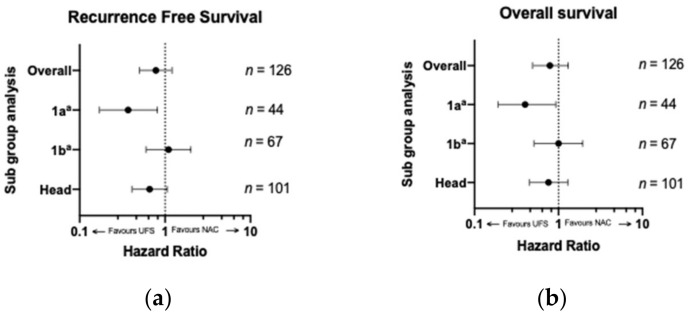
(**a**) Recurrence-free and (**b**) overall survival comparing UFS to NAC by subgroup analysis. Radiological stage 2a and tail were not included due to inadequate sample size. Legend: UFS—upfront surgery; NAC—neoadjuvant chemotherapy; ^a^—8th addition TNM staging system [21].

Sub-group analyses were pre-planned and included upfront stage and location of the tumor (Figure 3). Upfront stage 1a revealed a significant difference in median RFS in patients that received UFS at 18 months compared to 12 months for NAC (HR 0.30, *p* = 0.005; Figure 3a). This survival advantage also translated to an improved OS at 24 months for UFS compared to 21 months for NAC (HR 0.42, *p* = 0.03, Figure 3b). This difference was not reproducible for stage 1b for either RFS (*p* = 0.59) or OS (*p* = 0.97; Figure 3), and there were insufficient patients in the NAC group in stage 2a to allow for meaningful analyses (*n* = 2). There were 101 patients (38 NAC and 63 UFS) who had tumors arising from the head of the pancreas and 25 (2 NAC AND 23 UFS) had tumors arising from the tail. Median RFS for patients with head tumors was comparable (18 vs. 14 months, *p* = 0.09; Figure 3a), as was OS (24 vs. 19 months, *p* = 0.20; Figure 3b). There were only two patients in the body/tail subgroup that received NAC and analysis was not performed.

### 3.3. Surgical Outcomes

There was a statistically significant increase in complication rates at the time of surgery for patients who received NAC 19/38 (50%) compared to UFS 25/85 (29%) (chi-squared, *p* = 0.036). No difference between the two groups in the severity of complications could be appreciated (chi-squared, *p* = 0.256). Similar numbers of patients had R1 resections in both NAC 14/38 (37%) and UFS 36/85 (42%; *p* = 0.49). The rate of vein resection was significantly higher in NAC at 48% compared with UFS 18% (chi-squared, *p* = 0.001). Rates of perineural and vascular invasion between these two groups were also examined. In the NAC cohort 27/38 (71%) and 62/85 (73%) in the UFS cohort had perineural invasion and no difference between the two groups could be appreciated (chi-squared *p* = 0.97). There was, however, a significantly higher rate of vascular invasion (small or large vessel) in patients that underwent UFS at 68% (58/85) compared to 47% (18/38) of patients in the NAC cohort (chi-squared, *p* = 0.023).

### 3.4. Prognostic Factors

Factors identified as potentially prognostic after review of the literature are listed in Table 2. Both univariate and multivariate testing were performed. In multivariate analysis, no factors were identified that predicted for either recurrence-free or overall survival. Factors that were significant in univariate testing are discussed further.

#### 3.4.1. CA 19-9 and Imaging

Both baseline CA 19-9 (for all patients) and post NAC (for patients treated with NAC) CA 19-9 were collected. A cutoff of 500 kU/L was chosen as per international consensus to delineate low versus high probability of advanced disease [25]. In the NAC cohort, 30% (12/40) had CA 19-9 ≥ 500 kU/L compared to 18% (15/82) in the UFS. There was no difference in the percentage of patients with a CA19-9 above 500 kU/L in either group (chi-squared, *p* = 0.14). Baseline CA 19-9 ≥ 500 kU/L did not predict for either RFS (*p* = 0.33; Table 2) or OS (0.06; Table 2). All patients had a baseline CT abdomen for radiological staging. Upfront stage did not predict for RFS (*p* = 0.11; Table 2) or OS (*p* = 0.36; Table 2). Baseline FDG-PET scans were performed in 38/40 (95%) of the NAC and 35/86 (41%) of UFS cohort. FDG-PET was a useful prognostic marker with tumors that had a SUVmax ≥ 5 having a significantly shorter RFS at 14 months compared to 37 months in patients with SUVmax < 5 (HR 2.4, *p* = 0.02; Table 2). This difference was also evident in OS at 23 months in patients with SUVmax ≥ 5 compared to 51 months in patients with tumours SUVmax < 5 (HR 3.4, *p* = 0.007; Table 2).

#### 3.4.2. Histopathological Markers

At the time of pathological reporting an attempt was made to estimate the % of viable tumor cells remaining after surgery in patients that received NAC. The median viability was 80%. Higher tumor viability was associated with shorter RFS with an increase in hazard of 1.02 for every 1% rise in tumor viability (HR 1.02, *p* = 0.04; Table 2). This was also significant for OS (HR 1.03, *p* = 0.004; Table 2). For all patients, a higher tumor grade predicted for shorter RFS (HR 1.8, *p* = 0.005; Table 2) and OS (HR1.7, *p* = 0.017; Table 2). A higher T stage (size of the primary tumor) was associated with a decrease in OS (HR 1.6, *p* = 0.02; Table 2), however, had no impact on RFS (*p* = 0.12; Table 2). Patients with a higher nodal stage (increase number of nodes infiltrated with carcinoma) had a reduced RFS (HR1.4, *p* = 0.01; Table 2), however, this did not translate to change in OS (*p* = 0.05; Table 2). A positive margin (R1) predicted for shorter RFS (HR 1.5, *p* = 0.049), however not for OS (*p* = 0.21; Table 2). Vascular invasion was associated with shorter OS (HR 2.4, *p* = 0.001; Table 2), however not for RFS (*p* = 0.14) and perineural invasion did not predict for either RFS or OS (*p* = 0.18, 0.51; Table 2).

#### 3.4.3. Chemotherapy Regimen

Neoadjuvant and adjuvant chemotherapy regimens were matched for age. There were four neoadjuvant chemotherapy regimens utilized: gemcitabine; gemcitabine plus capecitabine; gemcitabine plus nab-paclitaxel; and folfirinox (Table A1). Thirty- seven patients received either gemcitabine-nab paclitaxel or folfirinox and there was no difference in RFS (*p* = 0.28) or OS between these two groups (*p* = 0.25; Table 2). The median duration of NAC was 8 (3–24) weeks. No difference in RFS (*p* = 0.29; Table 2) or OS (*p* = 0.26; Table 2) could be appreciated between patients who received ≥12 weeks of NAC compared to <12 weeks (Table 2).

Patients were more likely to commence adjuvant chemotherapy if they had an uncomplicated post-surgical recovery 65/78 (81%) compared to patients who had complications 32/45 (71%), however, this difference did not achieve significance (chi-squared, *p*= 0.11). In the NAC group, 25/38 (66%) of patients received further adjuvant chemotherapy compared to 69/85 (81%) in the UFS group. While adjuvant chemotherapy regimens were heterogeneous, the majority of patients received either gemcitabine (*n* = 36) or gemcitabine in combination with capecitabine (*n* = 24; Table A1). Patients who received gemcitabine plus capecitabine had a longer median OS compared to single agent gemcitabine therapy at 49 and 24 months, respectively (HR 0.6, *p* = 0.01; Table 2). There was no appreciable difference in RFS (*p* = 0.06; Table 2) between these two groups. The median duration of adjuvant chemotherapy was 24 (4–52) weeks [26,27]. Patients who received chemotherapy ≥24 weeks had a longer median RFS at 19 months (HR 0.5, *p* = 0.007; Table 2) compared to 12 months in those that had <24 weeks of chemotherapy. Similarly, there was an increase in OS for patients that received longer chemotherapy (≥24 weeks) at 49 compared to 20 months (HR 0.33, *p* = 0.0002; Table 2).

### 3.5. Predictive Markers of Chemotherapy Response in Patients Treated with NAC

Baseline and pre-operative CA 19-9, CT and FDG-PET were analyzed to explore their potential use as a marker to predict tumor viability, a surrogate for tumor response to chemotherapy. Serial CT scans (baseline and pre-operative) were performed on all forty patients undergoing NAC with a formal comparison report available for 33 of these patients. Using RECIST criteria four patients had progressive disease, 24 had stable disease, four had a partial response and one patient had a complete radiological response [18]. Similarly, baseline FDG-PET scans were performed on 38 of the 40 patients and again preoperatively in 22 of these patients. The standardized uptake value (SUVmax) was recorded for each patient and median on the baseline and pre-operative PET scan were 7.1 and 5.0 respectively. These radiological and biological markers were assessed for correlation to tumor viability on operative tumor specimen after NAC exposure (Table 3). Spearman rank analysis was used as all variables were considered non-parametric after Shapiro–Wilk testing.

Of these, only baseline SUVmax ≥ 5 (spearmans co-efficient, *p* = 0.023; Table 3) were useful in predicting higher tumor viability, and hence reduced chemotherapy response.

## 4. Discussion

In the context of recent randomized controlled trials PREP-02/JSAP-05 and PREOPANC-1 which reveal conflicting preliminary results, we conducted a retrospective cohort study and compared outcomes in patients diagnosed with resectable pancreatic adenocarcinoma who had been treated with either neoadjuvant chemotherapy (NAC) or upfront surgery (UFS) at one large pancreatic cancer center.

Our study revealed that for all stages, survival was comparable between the two cohorts; however, there was a statistically and clinically significant increase in RFS and OS in patients who had small, node-negative tumors (stage 1a) that received UFS. Furthermore, the proposed benefit of improved margin negative rates in patients who received NAC was not evident.

This improvement in survival in early-stage patients in our cohort treated with UFS makes biological sense. One of the purported benefits of NAC is that it allows exposure of potential micro-metastatic disease to cytotoxins early; however, the likelihood of disseminated disease in early tumors is lower than in advanced disease and the benefit in this context is as such diminished.

This study complements and expands on an earlier study conducted at our center. A retrospective study by Itchins et al. compared survival in patients with upfront and borderline resectable disease and found no survival difference between NAC and UFS [17]. Expanding on this study we aimed to compare NAC and UFS in patients with upfront resectable disease only, as the survival benefit of NAC over UFS in borderline disease has since been established [5,17,28]. Another strength of our study is that it has a larger number of patients with resectable disease who received NAC (*n* = 40) and outcomes were assessed using prospectively collected data. Although not performed on all patients, the widespread use of PET imaging and laparoscopy to rule out metastatic disease in particular prior to commencement of chemotherapy in our NAC cohort is also a strength. In the NEONAX trial 8% of patients in the NAC cohort were found to be metastatic at the time of surgery and an explanation given for this high number is that these patients may have had metastatic disease at the time of diagnosis that was not detected by routine imaging [29]. In our NAC cohort, 88% underwent a staging laparoscopy with negative peritoneal washings and 95% of patients underwent an FDG-PET making underdiagnosis of metastatic disease at diagnosis highly unlikely.

The importance of determining clinically useful biomarkers as surrogates for underlying tumor biology are becoming more prominent of late. At this stage, only CA19-9 is used clinically; however, more recent studies have identified additional potential biomarkers that if validated may prove an important aid in clinical decision making [30,31,32,33].

This is a hypothesis-generating analysis, which serves to highlight potential subgroups within the resectable population that may benefit from a particular upfront treatment modality and explore the role of markers identified in the literature in prognostication and prediction of chemotherapy response, within the confines of a retrospective review.

There are limitations to this study to note. This is a retrospective cohort study which introduces the potential of selection and recall bias. Although stages of the two groups were comparable it should be noted that the lack of randomization introduces the potential for selection bias toward NAC treatment for patients with more aggressive disease. The higher rate of vein resection in our neoadjuvant chemotherapy cohort indicate that this might have had more advanced disease at time of surgery. This may have been due to progression on neoadjuvant chemotherapy or a more advanced disease at diagnosis. The higher body/ tail lesions in the UFS group are a possible confounding factor and may resulted in a poorer survival in the UFS cohort [15,16] The higher rate of vein resection in our neoadjuvant chemotherapy cohort indicate that this might have had more advanced disease at time of surgery. This may have been due to progression on neoadjuvant chemotherapy or a more advanced disease at diagnosis. The higher body/ tail lesions in the UFS group are a possible confounding factor and may resulted in a poorer survival in the UFS cohort [15,16]. In addition, the lack of recorded Eastern Co-operative Group (ECOG) and co-morbidity status for all patients introduces a potential treatment selection bias. The surgical data is from a high-volume pancreatic cancer center by two pancreatic cancer specialist surgeons and surgical outcomes may not be representative of other centers around the world. In addition the high percentage of upfront surgical patients that received adjuvant chemotherapy (81%) is significantly higher than the 60% often reported in the literature [4].

## 5. Conclusions

This real-world study demonstrates that replacement of UFS with NAC as a standard of care for all patients with resectable disease may be premature. Further research in the form of a prospective trial is urgently required to compare these two upfront modalities with the incorporation of biomarkers to help to personalize the treatment of this devastating disease.

## Figures and Tables

**Table 1:** Baseline characteristics per upfront treatment modality.

	Neoadjuvant Chemotherapy	Upfront Surgery
Median age (years, range)	71 (42–85)	69 (41–90)
Sex		
F	20	53
M	20	33
Upfront radiological stage ^a^		
1a	12	32
1b	26	41
2a	2	5
2b		8
Total	40	86
Tumor location		
Head	38	63
Body/tail	2	23

F—Female, M—Male, ^a^ 8th addition TNM staging system [21].

**Table 2:** Univariate and multivariate prognostic markers for recurrence-free and overall survival.

RFS		Univariate		Multivariate	OS	Univariate		Multivariate
	*p*-Value	Hazard Ratio (HR) (95% CI)	*p*-Value	HR (95% CI)	*p*-Value	HR (95% CI)	*p*-Value	HR (95% CI)
Initial CA19-9 ≥ 500 kU/L	0.33	1.3 (0.77–2.19)	0.95	5.51 (0–5.6^21^)	0.06	1.7 (0.99–2.92)	0.63	3.6^6^ (0–3.28^33^)
Radiological stage	0.11	0.78 (0.58–1.06	0.95	0.18 (1.78^20^)	0.36	0.87 (0.64–1.2)	0.44	2.99^5^ (0–3.16^19^)
Viability (%)	* 0.04	1.02 (1–1.03)	0.79	0.99 (0.9–1.08)	* 0.004	1.03 (1.01–1.047)	0.92	1.07 (0.27–4.239)
Grade	* 0.005	1.8 (1.2–2.72)	0.58	1.7^9^ (2.7^41^)	* 0.017	1.7 (1.10–2.73)	0.29	5.86^12^ (0–2.57^36^)
Baseline FDG PET ≥ 5	* 0.02	2.4 (1.12–4.49)	0.88	0 (0–3.0^77^)	* 0.007	3.4 (1.40–8.33)	0.73	694 (0–1.78^19^)
Surgical stage T ^a^	0.12	1.3 (0.94–1.78)	0.81	0 (0–4.5^27^)	* 0.02	1.6 (1.08–2.48)	0.88	65.6 (0–7.42^24^)
Surgical stage N ^a^	* 0.01	1.4 (1.09–1.99)	0.86	9.6 (0–3.6^11^)	0.05	1.4 (1.00–1.95)	0.57	0.1 (0–9.24^4^)
Perineural invasion	0.18	1.4 (0.86–2.36)	0.79	1.8^4^ (0–8.8^35^)	0.51	1.2 (0.7–2.04)	0.38	1.19^16^ (0–2.04^52^)
Small/large vessel invasion	0.14	1.4 (0.89–2.16)	0.67	1.3^9^ (0–2.9^51^)	* 0.001	2.4 (1.4–4.08)	0.26	1.5^15^ (0–6.22^41^)
R1	* 0.049	1.5 (1.002–2.40)	0.73	75 (0–4.3^12^)	0.21	1.4 (0.84–2.1)	0.39	1.36^6^ (0–9.84^19^)
NAC GEM/ABR	0.28	0.65 (0.30–1.42)	0.88	2.6 (0–5.6^5^)	0.25	0.59 (0.24–1.45)	0.62	44.17 (0–1.5^8^)
NAC ≥ 12 weeks	0.29	0.95 (0.87–1.04)	0.98	0.20 (0–6.3^51^)	0.26	0.60 (0.25–1.46)	0.66	0 (0–1.79^18^)
AC GEM/CAPE	0.06	0.76 (0.58–1.01)	0.97	10.9 (0–1.4^48^)	* 0.01	0.60 (0.41–0.88)	0.74	2.6^3^ (0–4.48^23^)
AC ≥ 24 weeks	* 0.007	0.50 (0.30–0.82)	0.93	0.43 (0–1.16^30^)	* 0.0002	0.33 (0.19–0.59)	0.96	0.38 (0–6.6^10^)

HR—hazard ratio; RFS—recurrence-free survival; OS-overall survival; SUVmax—standardized maximum uptake value; NAC—neoadjuvant chemotherapy; GEM/ABR—gemcitabine and nab-paclitaxel; AC—adjuvant chemotherapy; GEM/CAPE—gemcitabine and capecitabine; R1—tumour within <1 mm at closet margin. * indicates statistical significance *p* < 0.05. ^a^ 8th addition TNM staging system [21].

**Table 3:** Biological and radiological predictors for tumor regression (tumor viability).

	*p*-Value	Correlation Coefficient (Spearmans)
CT response-RECIST ^a^	0.72	0.317
Baseline CA 19-9 ≥ 500	0.28	−0.18
Pre-operative CA 19-9 ≥ 500	0.32	−0.17
Initial SUVmax: ≥5	* 0.023	0.369
Pre-operative SUVmax: ≥5	0.45	0.175

SUVmax—standardized maximum uptake value. * Indicates statistical significance *p* < 0.05. ^a^ RECIST 1.1: Complete response (no evidence of primary lesion); partial response (reduction of >30% in the sum of the longest diameter of target lesion); progressive disease (≥20% increase in the sum of the longest diameter of target lesion); stable disease (does not fulfil criteria for complete response, partial response or progressive disease) [18].

## Data Availability

Data is available in the paper.

## Appendix A

**Table A1:** Type and duration of neoadjuvant and adjuvant chemotherapy.

Chemotherapy	Upfront Group: Neoadjuvant Chemotherapy	Upfront Group: Upfront Surgery
**Neoadjuvant chemotherapy**		
Folfirinox	12	N/A
Gemcitabine + Nab-Paclitaxel	25	
Gemcitabine + capecitabine	1	
Gemcitabine alone	2	
Median duration weeks (range)	8 (3–26)	N/A
**Adjuvant chemotherapy**		
Folfirinox		2
Gemcitabine + Nab-Paclitaxel	1	4
Gemcitabine + capecitabine	4	24
Gemcitabine alone	11	36
Capecitabine alone	9	1
S1		1
Unknown		1
Median duration weeks (range)	16 (3–24)	24 (4–52)

Number of patients receiving different chemotherapy regimens in each group in each group- both in the neoadjuvant and adjuvant setting.
